# QIN DAWG Validation of Gradient Nonlinearity Bias Correction Workflow for Quantitative Diffusion-Weighted Imaging in Multicenter Trials

**DOI:** 10.18383/j.tom.2016.00214

**Published:** 2016-12

**Authors:** Dariya I. Malyarenko, Lisa J. Wilmes, Lori R. Arlinghaus, Michael A. Jacobs, Wei Huang, Karl G. Helmer, Bachir Taouli, Thomas E. Yankeelov, David Newitt, Thomas L. Chenevert

**Affiliations:** 1 Department of Radiology, University of Michigan, Ann Arbor, Michigan;; 2 Department of Radiology and Biomedical Imaging, University of California San Francisco, San Francisco, California;; 3 Vanderbilt University (VU) Institute of Imaging Science, VU Medical Center, Nashville, Tennessee;; 4 Russel H. Morgan Department of Radiology and Radiological Science, John Hopkins University School of Medicine, Baltimore, Maryland;; 5 Advanced Imaging Research Center, Oregon Health and Science University, Portland, Oregon;; 6 Athinoula A. Martinos Center for Biomedical Imaging, Massachusetts General Hospital, Charlestown, Massachusetts;; 7 Translational and Molecular Imaging Institute, Icahn School of Medicine at Mt Sinai, New York, New York; and; 8 Department of Biomedical Engineering, University of Texas at Austin, Austin, Texas

**Keywords:** nonuniform diffusion weighting, gradient nonlinearity bias, correction validation

## Abstract

Previous research has shown that system-dependent gradient nonlinearity (GNL) introduces a significant spatial bias (nonuniformity) in apparent diffusion coefficient (ADC) maps. Here, the feasibility of centralized retrospective system-specific correction of GNL bias for quantitative diffusion-weighted imaging (DWI) in multi-site clinical trials is demonstrated across diverse scanners independent of the scanned object. Using corrector maps generated from system characterization by ice-water phantom measurement completed in the previous project phase, GNL bias correction was performed for test ADC measurements from an independent DWI phantom (room temperature agar) at two offset locations in the bore. The precomputed three-dimensional GNL correctors were retrospectively applied to test DWI scans by the central analysis site. The correction was blinded to reference DWI of the agar phantom at magnet isocenter where the GNL bias is negligible. The performance was evaluated from changes in ADC region of interest histogram statistics before and after correction with respect to the unbiased reference ADC values provided by sites. Both absolute error and nonuniformity of the ADC map induced by GNL (median, 12%; range, −35% to +10%) were substantially reduced by correction (7-fold in median and 3-fold in range). The residual ADC nonuniformity errors were attributed to measurement noise and other non-GNL sources. Correction of systematic GNL bias resulted in a 2-fold decrease in technical variability across scanners (down to site temperature range). The described validation of GNL bias correction marks progress toward implementation of this technology in multicenter trials that utilize quantitative DWI.

## Introduction

In the clinical cancer research community, there is growing interest in use of quantitative parametric maps for apparent diffusion coefficient (ADC) (1–3) derived from diffusion-weighted imaging (DWI) to assess tissue properties and evaluate therapeutic efficacy by monitoring changes in malignant tissue diffusivity (4–6). Because water mobility is expected to change depending on the density of cells in a tissue or tumor, by measuring the water mobility using DWI, information about tissue cellularity can be inferred (7, 8). A typical DWI acquisition is performed by applying additional diffusion gradient weighting, quantified by *b*-value. A widely used single-component diffusion model assumes monoexponential DWI signal decay with increasing *b*-value, where the decay rate is determined by the ADC value. To establish the diagnostic and clinical response benefits of quantitative DWI metrics for multicenter clinical trials, it is necessary to characterize and minimize systematic technical errors of DWI measurements (9, 10). Reduction of technical variability of ADC values could help reduce the required clinical trial size and lead to greater scientific fidelity (9, 10) of diffusion-derived biomarkers.

To address the technical variability in the quantitative DWI, the Data Acquisition Work Group (DAWG) of the National Cancer Institute (NCI) Quantitative Imaging Network (QIN) (11) launched a collaborative project to evaluate the impact of gradient nonlinearity (GNL) bias in diffusion weighting (DW) on ADC map values in multicenter trials, where the use of different scanner vendors and models (gradient systems) is inevitable. Using DWI measurements along the primary magnet axis for the temperature-controlled ice-water phantom (single ADC value), the DW GNL bias was characterized for individual gradient channels in 14 scanners with 10 distinct gradient systems (12). Significant ADC errors (ranging from +25% to −50%) were detected for a majority of systems (excluding one long-bore scanner). The GNL was found to be a major source of bias in the linear-offset DWI measurements, with minor contributions from shim and imaging gradient cross-terms (12). In fact, detected GNL bias accounted for about 95% of the observed absolute error in ADC value on a single magnetic resonance imaging (MRI) platform and resulted in an average 20% variation across MRI scanners. In contrast, excellent reproducibility was shown by multiple studies for the ice-water ADC measurements acquired at the magnet isocenter (12–14) (variability <3%).

The GNL-induced ADC errors scaled approximately quadratically with the distance from the magnet isocenter. Because this “scaling” was dependent on the gradient model, different ADC values would be measured on different gradient systems even for the same patient at the same location. On the other hand, the ADC value measured at a specific location on the same single system would deviate from the true value by a fixed absolute amount. Thus, when the same patient is repeatedly scanned at the same location with similar operator landmarking and positioning, the ADC map measurements would be repeatable (within measurement uncertainty), but biased by a fixed GNL error that varies as a function of location within the imaged volume. GNL bias would also artificially (nonbiologically) broaden the ADC histograms from large regions of interest (ROIs) of significant spatial extent along superior–inferior (SI), right–left (RL), and anterior–posterior (AP) directions (15). For multicenter clinical trials, when multiple patients are scanned at varying offset locations and/or on different scanners, “variable” ADC errors would be expected because of both different locations and different gradient system models.

In principle, when the system gradient properties are known (eg, from vendor (15, 16) or by empirical characterization (12, 17, 18)), these deterministic bias errors can be corrected (15, 18–20), and undesired technical variability can be reduced. That is, the GNL map is expected to be “static” over time for a specific gradient system. In fact, the same built-in system information is routinely used by vendors to correct for geometric distortions caused by GNL in spatial encoding via imaging gradients (17, 21, 22). However, corresponding GNL-related errors for DW gradients (*b*-values) remain uncorrected on current clinical scanners (12, 19, 20). In multisite clinical trial setting, the patient DWI scans are typically performed on multiple MRI platforms, whereas a single laboratory is charged with the centralized data analysis without open information on system-specific GNL characteristics. In the absence of “prospective” DW gradient correction on the scanners, empirical GNL maps can be obtained once (12, 17, 18) and used for “retrospective” correction of ADC maps (18) by individual sites and the central analysis laboratory. Minor non-GNL sources of DW bias, such as shim (chronic gradients adding to DWI gradients) and imaging cross-terms, would not be corrected by the sole removal of GNL bias, but could be mitigated by alternative methods (12), such as double spin-echo (DSE) DWI (23) or improved higher-order shim procedures.

In the present work, the empirical three-dimensional (3D) correctors generated from the original QIN–Data Acquisition Work Group GNL demonstration project (12) using an ice-water phantom were applied to independent function Biomedical Informatics Research Network (fBIRN) (24) phantom DWI scans performed for a subset of representative MRI scanners to show feasibility of the GNL bias removal in multicenter trials. The implemented two-phase study design imitated the typical multiplatform trial workflow, where proprietary information on gradient system characteristics is not available for a central analysis laboratory. The efficiency of correction was evaluated by comparing the ADC histogram metrics before and after the GNL correction with the reference (ground truth) ADC values that were measured by the individual participating sites from DWI phantom scans acquired near the magnet isocenter, nominally free of the GNL bias.

## Methodology

Six representative (precharacterized (12)) gradient systems (two from each vendor—Siemens, Erlangen, Germany; General Electric, Waukesha, Wisconsin; and Philips, Best, The Netherlands) were selected for validation of 3D GNL bias correction with an independent phantom. Gradient channel-specific numerical 3D GNL maps for these systems were constructed on the basis of previous ice-water phantom results (12) and applied for correction of new validation data.

**Figure 1.**
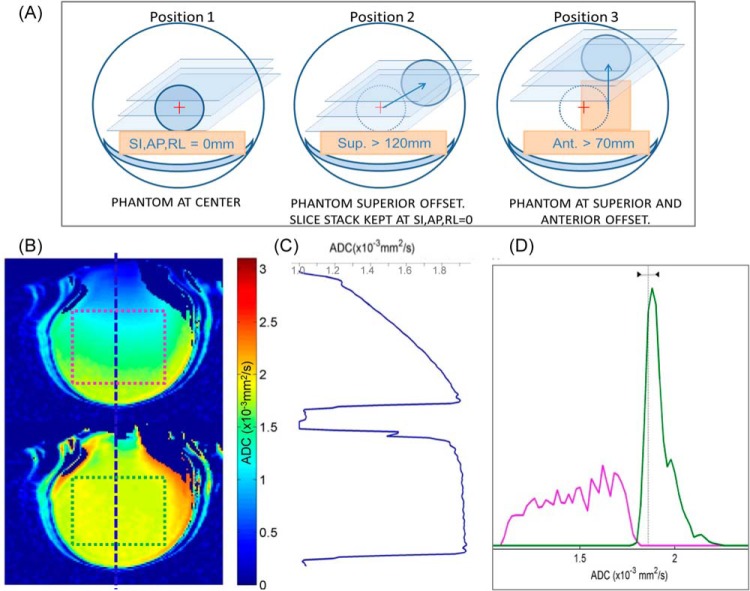
Schematic of fBIRN phantom positioning for GNL correction validation project (A). Conceptual example of phantom ADC map analysis for a gradient system before (top) and after (bottom) GNL correction using through-section (vertical blue dashed line) and in-plane (dashed rectangular) ROIs to quantify correction efficiency (B). A common scale for the ADC maps is indicated by the color bar. Sloped ADC profile (top) due to GNL bias along SI for linear ROI (B, blue line) is rectified by correction (bottom) (C). Correction efficiency is evaluated by proximity to reference scan ADC value measured by site (dashed vertical line), and restored uniformity of ADC map quantified by narrowing of the corrected histogram (green) compared to the original measurement (magenta) before correction (D).

The validation DWI scans were performed for the uniform gel fBIRN phantom at ambient temperature at three locations (as shown in Figure 1A) following the shared scan protocol (Supplemental Appendix). The uniform gel medium provided a single microscopic diffusion coefficient and prevented macroscopic swirling, although its absolute true diffusion value was dependent on local temperature and phantom age. The phantom was physically moved within a large field of view (FOV) keeping the original landmark. Position 1 (GNL bias free) scan data near magnet isocenter were kept onsite for reference ADC map measurements, whereas scan data for linear offset (superior), position 2 (with substantial predicted GNL bias >10%), and composite offset (superior–anterior), position 3 (with lower GNL bias <10%), were submitted for centralized analysis and correction. The sites were instructed to independently measure and record the reference ADC values and standard deviation (SD) on their scanner consoles using manually defined cubic ROIs, extending less than ±15 mm from the isocenter to minimize GNL bias in reference ADC values.

Because the focus of the study was on validation of pure GNL correction, the experimental acquisition protocol was designed to mitigate the non-GNL contributions to ADC bias (such as shim quality) as much as possible (12) (eg, by using DSE DWI (23), if available on a given system, and the best local shim procedure). Multiple averages and a relatively course imaging grid with subsequent zero-fill interpolation were prescribed to boost the signal-to-noise ratio (SNR) such that the random measurement errors quantified by the DWI SD in the studied *b*-value range were lower than the predicted systematic GNL bias at offset positions. All acquired data were stored in Digital Image Communication in Medicine (DICOM) format (25), and the centralized analysis of positions 2 and 3 DWI DICOM data was automated using routines developed in Matlab 7 (The MathWorks, Natick, Massachusetts).

### DWI of FBIRN Phantom

Each participating site scanned their own fBIRN phantom (24) consisting of a 17-cm-diameter plastic sphere filled uniformly with 1.5% agar (in water) gel medium at ambient temperature. The sites retrospectively recorded their scanner bore temperatures (±1°C), and the reported values ranged between 17°C and 22°C among sites. Based on the known temperature dependence of water diffusion coefficient (2.5% per degree, (26)), the predicted range of thermal ADC variations among sites was 10%–15%.

Coronal 2D DWI scans for 25–29 slices were acquired using single-shot echo-planar imaging (EPI) and three *b*-values (*b* = 0, 500, and 1000 s/mm^2^) for three orthogonal DWI directions and eight excitations per *b*-value. The acquired DWI had SNR >15 for any single direction of the highest *b* = 1000 s/mm^2^. Additional key acquisition parameters that varied among systems are listed in Table 1. Echo time (TE) ranged from 80 to 120 milliseconds. Most scanners used an orthogonal DWI gradient schema along the primary magnet axes (“LAB”):$U=\left[{\left(1,\;0,\;0\right)}^{T},\;{\left(0,\;1,\;0\right)}^{T},\;{\left(0,\;0,\;1\right)}^{T}\right]$, and systems labeled “PH1” and “PH2” acquired additional “non-LAB” DWI data using: $U=\left[{\left(-1/3,-2/3,-2/3\right)}^{T},\;{\left(2/3,-2/3,1/3\right)}^{T},\;{\left(2/3,1/3,-2/3\right)}^{T}\right]$. The DWI gradient directions and acquisition parameters were automatically extracted from DICOM headers. Three systems used the DSE sequence variant. The 1.5 T systems used only first-order shim. Volume shim methods used by scanners differed mainly in extension of the rectangular shim boxes that included arbitrary portions of phantom and air. One system, “PH1,” used a 10-cm shim box that led to excessive local shim gradients in the vicinity of the box (and FOV) edges, not corrected by performed single spin-echo (SSE) DWI acquisition (12). The DWI images for position 3 from the systems labeled “SM1” and “SM2” were submitted with acquired resolution and were interpolated to 256 × 256 by the central analysis site. The same two systems had limited FOV (448 mm), with severe image distortions near the edges that precluded larger phantom offsets for positions 2 and 3 scans. No geometric correction for EPI image distortion was used.

**Table 1.** Key Acquisition Parameters and Generic Model Scale-Factors for Studied Gradient Systems

Parameter/System	PH1	PH2	GE1	GE2	SM1	SM2
Manufacturer	Philips	Philips	GE	GE	Siemens	Siemens
Model	Achieva	Ingenia	Signa Whole	Signa Zoom	Skyra	Espree
Magnetic field	3 T	3 T	1.5 T	1.5 T	3 T	1.5 T
Field-of-view	500	500	500	500	448	448
Slice thickness/gap	5/1	6/1	5/1	5/1	5/1	7/1.4
Acquisition matrix	123 × 123	100 × 100	128 × 128	128 × 128	100 × 100	90 × 90
TE (ms)	95 (84)^a^	88	108	90	117	120
TR (s)	5.32	4.00	8.30	8.30	5.00	5.00
DWI sequence	SSE	SSE	SSE	DSE	DSE	DSE
Shim box (cm)	10 × 10	17 × 17	17 × 17	17 × 17	37 × 31	38 × 28
*L_xx_* model scale	1	1.25	1.2	4	1.1	2
*L_yy_* model scale	1	1.15	1.2	2.2	1.2	1.9
*L_zz_* model scale	0.7	0.7	0.2	0.45	0.9	0.9

Abbreviations: SSE, single spin-echo; DSE double spin-echo; DWI, diffusion-weighted imaging.

^a^ TE for “non-LAB” DWI acquisition.

### Systematic GNL Bias Prediction and Correction

The 3D GNL maps for individual gradient channels were empirically derived, with methods similar to Malyarenko et al.'s (2014) study (18) based on previous channel-specific DWI measurements, performed as a function of an offset from the isocenter (±155 mm) for an ice-water (tube) phantom accurately aligned along the RL and SI primary magnet axes (12). In brief, using spherical harmonics' coefficients provided in Janke et al.'s study (17), the “generic” numerical 3D model was constructed on a 5-mm grid within FOV = 600 × 600 × 600 mm^3^ for 9 components of the GNL tensor, ${\text{L}}^{M}\left(r\right)$ (19). Because the ***L^M^*** value range was inadequate for describing several gradient channel measurements in our previous phase one study (12), the value scaling (in contrast to spatial scaling (18)) was performed for all systems to generate 3D maps for their corresponding nonlinearity tensors, ***L***(***r***). The GNL model scale factors, *F*_*i*_, for each individual gradient channel, *i* = *AP, RL, SI*, were obtained by simultaneously fitting the linear SI and RL cross sections of diagonal GNL terms for the rescaled generic model, $L_{ii}\approx F_{i}\;\cdot \left(L_{ii}^{M}-1\right)+1$ to the corresponding empirical ADC map measurements (12), $L_{ii}\approx \sqrt{ADC_{i}\text{/1.1}}$. Typical model fit, *F*_*i*_, errors ranged between 3% and 7%, depending on the original measurement error (12) and the ability to fit single scaling factors adequate for both SI and RL(AP) directions (18). In addition to the scaling of diagonal nonlinearity tensor components, $L_{ii}\left(r\right)$, the off-diagonal tensor elements (not measured directly) were rescaled as $L_{ij}\approx F_{i}\;\cdot \;L_{ij}^{M}$. The used system-specific GNL range-scaling factors are summarized in Table 1. Once constructed, the rescaled system-specific nonlinearity tensors remained fixed for further analysis and correction of fBIRN DWI data. Three systems also provided vendor design GNL information.

Using the rescaled (or design) nonlinearity tensor, for each DWI gradient direction, ***u***_*k*_, the predicted DW (*b*-value) bias (corrector) maps were generated as a Frobenius norm of a dyadic product, $C_{k}=\|Lu_{k}{\left(Lu_{k}\right)}^{T}\|$ (15, 20). For DWI correction in the isotropic fBIRN phantom, a single direction-average corrector map, *C_av_*, was constructed for the applied orthogonal DWI ***U***-schema (“LAB” and “non-LAB”) and interpolated according to experimental DICOM header information on image volume and resolution. The predicted bias was symmetrically lower along SI (negative GNL bias) and higher along RL/AP (positive GNL bias) than nominal (isocenter *C_av_* = 1, where GNL was absent), leading to correspondingly under- or over-estimated ADC values (eg, Figure 1, B and C, top). A corrected ADC map was derived from pixel-by-pixel division of “measured” ADC map by the corresponding corrector (15, 18) as follows: $ADC^{c}\left(r\right)=ADC\left(r\right)/C_{av}\left(r\right)$ (eg, Figure 1, B and C, Bottom). For completeness, the alternative corrections of trace DWI image intensities and *b*-values (15, 20) were likewise performed and found to be nominally identical to the direct ADC map correction, as expected for an isotropic diffusion medium in a uniform gel phantom.

The applied correction did not account for geometric distortion due to EPI, which limited correction accuracy for the image pixels with the steep GNL “gradients” ($dC_{av}/dr$), when the apparent pixel location is shifted from its true position assumed for $C_{av}\left(r\right)$. For the system with the largest GNL scales (“GE2” and “SM2” in Table 1), the error due to geometric distortion predicted for GNL correction at the remote phantom offsets (>120 mm) used by a current protocol was up to 5%. The derived correctors did not include contribution from chronic (or local) shim gradients (12).

### ADC Map ROI Analysis and Evaluation of Correction Efficiency

Centralized analysis of phantom data for positions 2 and 3 was performed by a single site to generate the ADC maps (eg, Figure 1B) from native DWI DICOM as a difference between log-trace DWI (direction-average image) pixel intensities for the high *b*-value and *b* = 0 divided by the high *b*-value. The calculated ADC values outside the range of 0.5–3.3 (×10^−3^ mm^2^/s) were set to zero. The GNL bias correction performance was evaluated by comparison of ADC ROI histogram statistics before and after correction to the reference values measured by sites (eg, Figure 1D). Original GNL nonuniformity within a relatively large ROI resulted in an artificial broadening of the ADC histogram accompanied by a shift of the mean/median ADC value (eg, Figure 1D, magenta). For meaningful comparison of histogram statistics across systems, histogram ROI locations were selected on slices at comparable AP elevation (position 2, 5–7 mm; position 3, 80–100 mm). For the selected position 3 ROIs, by the physical nature of horizontal-bore nonlinearity, the negative GNL bias along the SI axis is partially offset by the positive bias along the AP axis, reducing overall GNL-induced nonuniformity of ADC maps. This effectively provided a “negative” control for the GNL correction and test for non-GNL sources of ADC nonuniformity.

The rectangular section ROI was defined by selecting SI and RL pixel ranges that spanned the full image portion that was relatively free of severe distortion or artifacts (eg, Figure 1B). The ROI centroids and volumes used for the analysis are listed in Table 2. Although the selected SI and RL ranges varied among systems, depending on image distortion (apparent deviation from spherical shape), comparable spatial areas/volumes were used (Table 2: P(volume 2 vs. volume 3) = 0.39). This required splicing the available data for “SM1” and “SM2” systems from two adjacent slices because of excessive distortion of more than half of their individual slice images.

**Table 2.** Summary of ADC ROI Histogram Statistics for FBIRN Positions 2 and 3 before (“pre”) and after (“post”) Correction of System GNL Bias and for the Reference Scan

Metrics⃥System	PH1	PH2	GE1	GE2	SM1	SM2
“Position 2” ROI						
Center (AP, RL, SI)^a^	(6, 2, 123)	(7, 26, 113)	(5, 12, 94)	(7, 1, 86)	(7, −28, 90)	(3, −18, 84)
Volume (cm^3^)	41	53	41	46	55	43
“pre” median (ADC)^b^	1.65	1.67	1.72	1.55	1.88	1.80
“pre” FWHM^b^	0.32	0.35	0.24	0.45	0.21	0.31
“post” median (ADC)	1.96	1.95	1.89	1.89	2.06	1.96
“post” FWHM	0.11	0.08	0.11	0.08	0.15	0.14
“Position 3” ROI						
Center (AP, RL, SI)	(−92, 16, 123)	(−96, 99, 99)	(−92, 16, 104)	(−93, 4, 95)	(−91, −45, 45)	(−86, 13, 79)
Volume (cm^3^)	41	54	34	42	37	49
“pre” median (ADC)	1.87	2.04	1.84	1.83	2.08	2.12
“pre” FWHM	0.13	0.16	0.11	0.14	0.20	0.27
“post” median (ADC)	2.08	1.96	1.92	1.91	2.16	2.08
“post” FWHM	0.18	0.08	0.10	0.11	0.15	0.16
“Reference” ROI						
Center (AP, RL, SI)	(0, 0, 0)	(0, 0, 0)	(−1, −8, 0)	(0, −1, 0)	(2, −2, 0)	(3, 0, 0)
Volume (cm^3^)	32	33	27	27	32	34
Median (ADC)	1.93	1.95	1.83	1.86	2.11	1.95
FWHM	0.05	0.06	0.06	0.05	0.1	0.08

^a^ROI center coordinates are in “mm”.

^b^ADC median and FWHM (CI = ±0.02) are in units of “10^−3^ mm^2^/s”.

The sites performed independent reference ADC value and SD measurement on a scanner console from a 30 × 30 × 30 mm^3^ ROI centered at the isocenter (which spanned 5 slices) to sample a sufficient volume with minimal expected GNL bias and distortions. Because of limited spatial extent of a GNL-free region, reference ROI volume was significantly smaller than the ROI volume for either position 2 (*P* = 2E-3) or position 3 (*P* = 4E-3). Three sites also exported corresponding reference ADC histograms for off-line analysis. GNL corrections were performed and finalized by the central processing site without knowledge of reference ADC values generated at acquisition sites.

The ADC map ROI histograms were binned with a step of 0.02 between 1 and 2.5 (×10^−3^ mm^2^/s). The median and full-width at half-maximum (FWHM) ranges were chosen as histogram metrics due to non-Gaussian character of broadening induced by the GNL bias (15, 20). In the absence of the GNL bias (eg, reference ADC), these metrics were equivalent to mean and 2SD (95% confidence interval (CI)) of Gaussian distribution dominated by ROI noise. Histogram nonuniformity was quantified by the FWHM-to-median ADC value ratio. Histogram width in excess of ROI noise (95% CI) was interpreted as a quantitative measure of the bias-induced nonuniformity of ADC maps (both from GNL and non-GNL sources). Reduction in nonuniformity and the proximity of median values before and after correction to the reference ADC (eg, Figure 1D), quantified by percent bias, %(ADC − ADC_ref_)/ADC_ref_, were used as figures of merit for GNL bias correction. Variability across systems was characterized by the range-to-median ratio. Wilcoxon rank sum test, P, was applied to find significant changes in histogram statistics. The Pearson correlation coefficient, R, across systems was calculated to reveal the dependencies between histogram metrics (Table 2) and acquisition parameters (Table 1).

## Results

**Figure 2.**
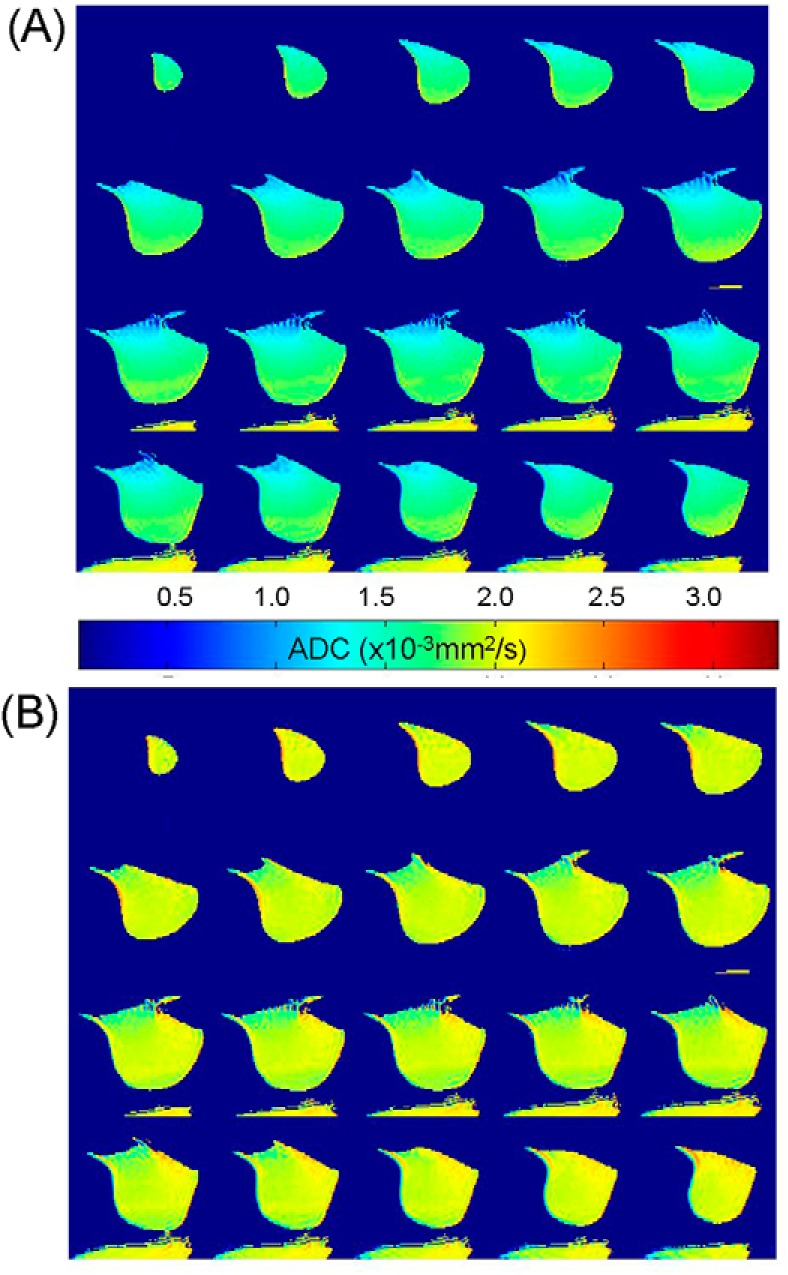
Example of 20-slice montage for an ADC map of fBIRN phantom from the “PH1” system with “non-LAB” DWI acquired at position 2 is shown before (A) and after (B) GNL bias correction to illustrate restored uniformity of the ADC map throughout the phantom volume. Common ADC scale for (A) and (B) is indicated by the color bar. The extra signal below fBIRN phantom observed on some images is from the water bottle placed at the FOV center to facilitate tuning.

According to the study design, position 2 data with GNL bias exceeding non-GNL sources were the most informative regarding the efficacy and feasibility of the GNL correction, whereas position 3 data, with minor GNL error, were used as a baseline for non-GNL contributions. Figure 2 shows how the GNL bias correction based on the precomputed (static) nonlinearity maps restores ADC uniformity within the full imaged 3D volume for the independent phantom measurement (at 12-cm SI offset with “non-LAB” DWI schema) for the “PH1” system. The nonuniformity range before correction reflects both intrinsic system GNL properties and (mainly) SI range covered by the selected section ROI (Table 2). The color gradient toward decreasing ADC along the SI direction induced by GNL bias is visually rectified by correction, shifting, and reducing the original ADC (×10^−3^ mm^2^/s) range of 1.3–1.9 (color bar) to a much more uniform range of 1.9–2.0. The residual ADC nonuniformity is mainly driven by the measurement noise, non-GNL bias contribution, and GNL model uncertainty. Minor amplifications of residual nonuniformity evident for the areas with pronounced geometric distortion (∼10 mm) are related to both uncorrected contribution of shim bias (<5%) for the SSE acquisition and finite errors (<3% for “PH1”) of the local GNL correction that uses correction factors at apparent (rather than true) locations (Methodology). Notwithstanding the aforementioned limitations, the achieved substantial improvement of ADC uniformity over the imaged volume confirms the feasibility of successful extension of the empirical GNL model, based on limited-range (linear) measurements and “LAB” DWI, to arbitrary spatial volume and DWI directions. Similar results were obtained for all studied gradient systems.

**Figure 3.**
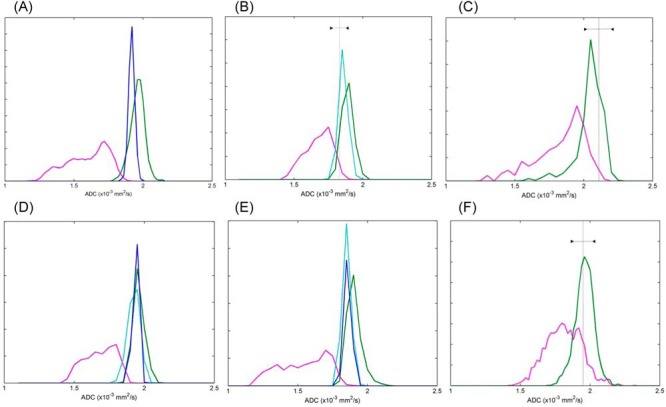
Position 2 ADC ROI histograms (bin size, 0.05 × 10^−3^ mm^2^/s) are shown for all systems: “PH1,” “GE1,” and “SM1” (in A–C) and “PH2,” “GE2,” and “SM2” (in D–F). The corresponding ROI locations are listed in Table 2. Magenta histograms indicate original ADC measurements (before correction) and green histograms denote corrected ADC maps, using generic (scaled) GNL models (as described in Methodology). Results of correction based on vendor-provided system GNL models are overplotted (where available) as dashed cyan traces in (B), (D), and (E). Reference ADC histograms, exported offline by several sites, are shown in blue in (A), (D), and (E), and reference values with corresponding 95% CI, independently measured on scanner console by other sites, are shown as vertical and horizontal black dotted lines, respectively.

Figure 3 and Table 2 provide a quantitative summary of the position 2 ADC correction results across systems for rectangular ROI histograms on the section near the 6 mm AP offset. For all gradient systems, the corrected histograms (green) were narrowed at least by half compared to the original ones (magenta), and their medians were brought within the 95% CIs of the corresponding reference measurements (dashed black lines). The overall shape of the histograms changed from skewed, non-Gaussian to more Gaussian-like after correction, consistent with reduction of nonrandom (systematic) bias noise. For the two systems that performed both “non-LAB” and “LAB” DWI, the original bias and correction efficiency were relatively independent of DWI direction schema (histograms not shown). Small residual offsets observed for some corrected histograms from their corresponding references (eg, Figure 3, A, B, and E) were likely related to the limited precision of the approximate (empirical) GNL models compared with the actual gradient system characteristics. The use of vendor-provided spherical harmonics' coefficients for GNL description of “GE1” and “GE2” systems (Figure 3, B and E) further improved corrected histogram alignment with the reference. Residual width of corrected histograms in excess of reference CI likely reported on the contribution of non-GNL sources: e.g., shim bias for SSE systems in Figure 3, A, B, and D and geometric distortion for DSE systems in Figure 3, C, E, and F. These contributions are amplified at remote locations compared with reference measurement near the isocenter, and are not expected to be corrected by the sole removal of the GNL bias.

The original differences among all systems in ADC (×10^−3^mm^2^/s) ROI histogram metrics (Table 2) for position 2 (range/median = 0.33/1.69) were substantially reduced by the GNL correction (0.17/1.91), indicating effective 2-fold suppression of bias-induced (technical) variability (from 20% to 9%). The variation among sites in “reference” ADC values (0.28/1.94) measured for position 1 was not significantly correlated with the original position 2 ADC value variation observed before correction (R = 0.72, *P* = .1), likely obscured by system-dependent GNL bias. The GNL correction restored correlation with the median ADC values for position 2 to the reference (R = 0.99, *P* = 1.E-4), indicating similarity between residual cross-site variability of corrected ADC and original site dependence of the reference ADC values. Analogously, restored correlation with the reference ADC, albeit to a lesser degree (R = 0.85, *P* = .03), was observed for corrected position 3 data. The observed reduction in the degree of similarity (0.85 < 0.99) compared with that in position 2 was consistent with additional (uncorrected) contribution from non-GNL sources that were likely present for position 3 (eg, different local shim gradients and geometric distortions). The 14% range/median for reference ADC values reported by sites was within the limits predicted from thermal variations because of the 5°C range of the scanner bore temperatures (Methodology).

For position 2 ADC ROI (Figure 3, Table 2), the histogram FWHM (in 10^−3^ mm^2^/s) across sites before correction (median, 0.32; range, 0.21–0.45) was significantly (*P* = 2.2E-3) reduced by correction (median, 0.11; range, 0.08–0.15). In contrast, no significant (*P* = .3) FWHM reduction was observed for position 3 ROI before (0.15; 0.11–0.27) versus after GNL correction (0.11; 0.08–0.18), consistent with the experimental design, whereby GNL was a minor source of bias for this location. The fact that residual broadening errors after correction were comparable for positions 3 and 2 (with substantially different initial contribution from GNL-induced bias) indirectly confirmed the non-GNL source of the residual errors. The original histogram width before correction for “SM1” and “SM2” was apparently lower (compared with other systems) because of the limited artifact-free phantom ROI area used (see Methodology), although their model GNL characteristics were comparable to other systems (Table 1, scale factors).

The median FWHM (CI) for site reference scans was 0.06 (range, 0.05–0.1) × 10^−3^mm^2^/s, with narrower CIs significantly correlated with shorter scan echo times (R = 0.83, *P* = .04) and thus to higher SNR. This correlation provided indirect evidence that reference CIs were mostly driven by random noise within an ROI. Residual ADC histogram FWHM after GNL correction exceeded the reference CIs about 2-fold, likely because of both inaccuracies of the system GNL model approximation and a finite contribution from non-GNL bias sources (eg, shim errors for SSE acquisition and image distortion for DSE acquisition).

**Figure 4.**
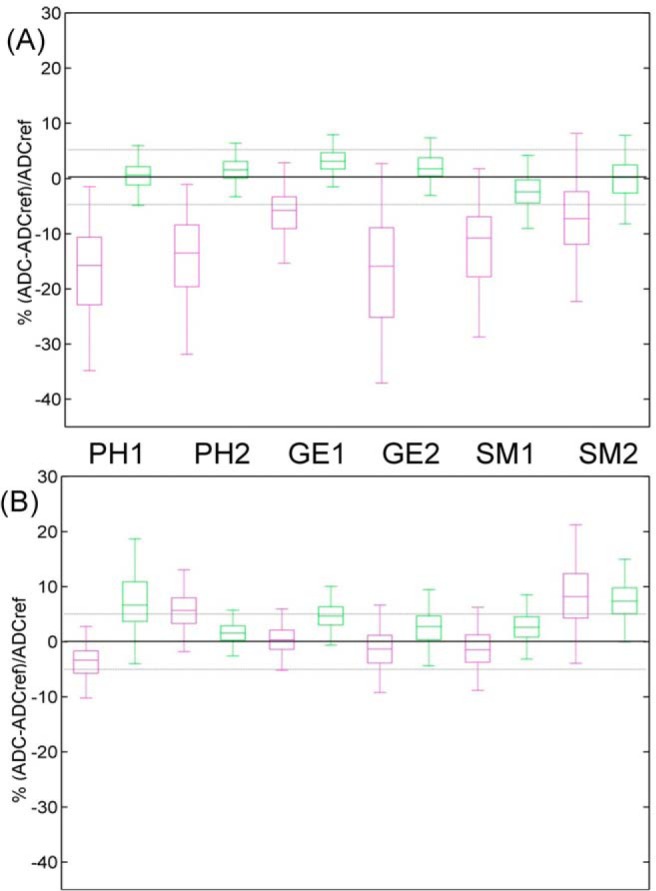
Percent-bias box plot summary for ADC ROI histograms at position 2 (A) and position 3 (B) measured for all systems before (magenta) and after (green) GNL bias correction. The corresponding ROI locations and statistics are listed in Table 2. Median percent-bias values are marked with the central line inside the box. The edges of the box correspond to 25th and 75th percentiles, whereas whiskers encompass the 5th–95th percentile data points. The dashed horizontal lines delineate ±5% error ranges.

To quantify correction efficiency across systems, the summary of percent bias metrics with respect to reference ADC values for both phantom locations (positions 2 and 3) is shown in Figure 4 in a box-plot format. The ±5% range (dashed horizontal lines) envelopes the ROI noise level corresponding to both the average GNL model fit uncertainty (Methodology) and the highest CI for the system-wide reference ADC (median, 3.2%; range, 2.6%–4.8%). While Figure 4A is effectively a dimensionless summary of percent differences between Figure 3 histogram metrics and reference ADC values (Table 2), Figure 4B shows the results for the position 3 ROI slice offset anteriorly by about 10 cm from the position 2 slice ROI (Table 2), where original GNL-induced bias is small.

The ADC errors for the position 2 ROI (Figure 4A) substantially exceed (up to −25% bias for the 75th percentile) the ROI noise and model fit uncertainty level of ±5% (dashed lines) before correction; however, their 25–75th percentile remained mostly within the reference CI after the GNL correction. The corresponding median values (−16% to −6%) and 95th percentile data ranges (−35% to +10%) for all systems moved significantly closer (*P* = 2.2E-3) to the reference (Figure 4A, zero-error line) after correction (medians, −3% to +3%; 95th percentile ranges, −9% to +8%). The percent range-to-median absolute error across systems was reduced from 12% (range, 6%–17%) before correction to 1.6% (range, 0.5%–3.3%) after correction. As expected (see Methodology), for the position 3 ROI, the ADC bias errors before correction were less conspicuous (median, 3%; range, 0.6%–8.7%) with no evidence (Figure 4B) of significant change (*P* = .3) in ADC error ranges after correction (median, 3.8%; range, 0.5%–7.7%).

The percent nonuniformity error (FWHM/median) significantly reduced (*P* = 2.2E-3) for position 2, from median of 18.2% (range, 11.2%–29%) before correction to 5.7% (4.1%–7.3%) after GNL bias correction. As expected, no significant (*P* = .18) reduction was observed for (mainly) non-GNL-induced nonuniformity of position 3 ADC before 8.1% (6.4%–12.7%) versus after 6.4% (4.1%–8.7%) GNL correction. Comparison of residual nonuniformity error with the corresponding noise-induced nonuniformity of the reference ADC values of 3.2% (2.6%–4.8%) indicated that about a half (∼2%–5%) of the residual nonuniformity after correction (for both positions 2 and 3) was still attributed to systematic bias sources (eg, chronic shim gradients and image distortion).

Notably, for 2 outlier systems in Figure 4B (labeled “PH1” and “SM2”), the corrected median ADC values were above the random noise level, indicating an apparent “overcorrection” of the GNL bias for “PH1” and “undercorrection” for “SM2”. Closer investigation of the ADC map artifacts for the “PH1” system revealed that the observed overcorrection was likely related to the non-negligible shim bias contribution to *b*-value from local chronic gradients (not corrected by applied SSE acquisition) caused by site usage of the shim-box size smaller than the phantom diameter (Table 1). The presence of uncorrected (regional) shim gradients was also evident from the RL flip of the in-plane shim distortion direction through different AP slice locations (Figure 2). For position 3 ROI, close to the shim-box edges, the local shim gradients counteracted the small effects of GNL bias, leading to apparent overcorrection of ADC nonuniformity (and increased corrected histogram width) for the “PH1” system. In contrast, for the “SM2” outlier, the GNL bias correction improves histogram nonuniformity, but fails to bring the residual errors down to reference CI level. This behavior is indicative of the limitation of the applied empirical GNL model for the composite anterior–superior offset compared with pure superior offset for position 2 (Figure 4A). The corresponding model error likely stemmed from different spatial scale factors of AP and SI GNL required for more precise “SM2” system description, whereas a single scale was used in the current approximation (Methodology). In such cases, further reduction of systematic error in ADC maps would require more precise GNL model approximation and shim bias correction.

## Discussion

Our study illustrates the feasibility of practical implementation of the two-phase workflow for the centralized retrospective correction of the GNL bias in DW gradients in the context of a multicenter clinical trial using an ADC map-based biomarker. Static GNL corrector maps that are theoretically independent of the imaged object were calculated once for a system-specific model based on previously acquired empiric data from a temperature-controlled DWI phantom. In the current phase of the workflow, these extrapolated system-specific 3D corrector maps were successfully applied to DWI scan data of an independent object at arbitrary offset locations in the magnet bore. Two locations were chosen (one with significant and one with minor GNL bias contribution) to quantify GNL correction efficiency and residual baseline ADC error from non-GNL sources across scanner systems. Consistent with previous findings (12–14), GNL caused nonuniform DW that followed spatially static patterns for a given system, independent of the nominal *b*-value and applied three-orthogonal DWI direction schema, and was readily predictable from the deterministic gradient channel characteristics (15, 16, 19, 20). This deterministic property enables implementation of practical GNL bias correction in multicenter setting by first obtaining empirical system characteristics through one-time phantom measurements and subsequently correcting *b*-values for this system before generating ADC maps for cross-site analysis.

Our results confirm the longitudinal stability of system GNL characteristics as evident from successful application of static correctors obtained based on three-year-old measurements (12) to newly acquired independent data. For phantom position 2, where GNL bias was a major contributor to the ADC error, the applied correction substantially (3-fold) improved the uniformity of the ADC maps in 3D for all the systems, removed up to 30% absolute ADC bias, reducing absolute error with respect to reference >7-fold, and suppressed technical cross-system variability >2-fold. The residual ADC nonuniformity errors (4%–8%) were down to the level of (uncorrected) non-GNL bias and measurement noise CI, and had about equal contributions from these sources. Elimination of non-GNL bias sources, not addressed by this study, would be desirable to ensure further reduction of ADC nonuniformity errors down to the measurement uncertainty level.

The median ADC map range across systems after correction (∼9%) reflected (5°C) temperature range of the scanner bores, whereas much higher variability (∼20%) before correction was half-driven by the scanner-specific GNL bias. Site-dependence of corrected ADC values was effectively restored to a level similar to that observed for the “reference” scans obtained at the magnet isocenter. Relatively large (14% range) thermal variations in reference ADC values observed among sites confirmed the importance of a temperature-controlled phantom for precise empirical system GNL characterization (performed in phase one (12)). The observed thermal-induced variability issue was relevant for phantom (validation) study only, and is not expected to affect clinical trial application, where the subject's body temperature is naturally regulated. For this application, elimination of GNL-induced bias across scanner systems would clearly remove the major source of technical (nonbiological) variation.

The analysis of residual nonuniformity for baseline position 3 with small GNL bias contribution revealed the following three possible sources of residual systematic errors (in addition to measurement noise) after GNL correction: (a) imperfect empiric approximation (3D extrapolation) of the actual system GNL, (b) non-negligible contribution of the “second-order” effects, e.g., when some spatial locations have large regional shim errors, and (c) all true second-order contributions not correctable by GNL (including EPI distortion in steep GNL regions).

Elimination of sources (b) and (c) was outside the scope of this work (other than by evaluation and suppression through scan protocol adjustment). However, our results suggest that these sources should be (at minimum) monitored by independent mapping (eg, of local field gradient distortions), and their impact should be minimized when possible by using the second-order volume-tailored shim and/or DSE DWI, as well as image coregistration. For body DWI applications focused on torso organs, regional (cylindrical geometry) shim bias is expected to be insignificant, as was shown for rectangular flood phantom (15). More rigorous studies of local shim errors for spherical geometry (eg, fBIRN phantom) would require a focused design to evaluate different shim parameter combinations with DSE and SSE sequence variants and are deferred to future research.

The known source (a) limitations of the empiric GNL model-rescaling approach adopted in this work stem from the following three approximations: (1) 1D scaling factors are measured with finite uncertainty, (2) a single scaling factor accounts for both RL/AP and SI nonlinearity over a large 3D volume (18), and (3) cross-channel GNL contributions have the scale of one of the channels. These limitations can be potentially overcome by comprehensive empirical system characterization in 3D, using field cameras or geometric distortion phantoms (17), complemented by the usage of proprietary gradient system design coefficients, known to MRI vendors, to analytically derive system-specific full 3D GNL corrector functions for DWI gradients (15, 16). Ideally, in complete analogy to prospective correction of GNL spatial distortions currently performed on clinical MRI systems, integrated “on-scanner” implementation of GNL bias correction for DWI could use vendor-provided correction routines locally run on an MRI system before image export. This preferred correction venue is currently being explored through the academic industrial partnership with three dominant MRI manufactures by three QIN centers that participated in the described GNL-correction validation project.

In conclusion, this work shows that centralized retrospective correction of GNL bias in DW, obtained from one-time empiric characterization of system GNL, is warranted by the stability of gradient channel characteristics, is desired for substantial reduction of ADC map bias, and is clearly feasible in multicenter clinical trial setting. In the absence of the preferred, prospective GNL correction using system design coefficients or complete independent 3D gradient field mapping, available (approximate) empirical correctors provide a practical solution for substantial improvement by removing systematic nonuniformity bias at off-center locations and reducing technical variability across multiple scanner systems. When not corrected, this technical bias both shifts and artificially broadens the corresponding ADC ROI histograms, and increases cross-system variability of the quantitative DWI metrics. The reduction of systematic ADC map errors using the proposed technology will have a positive impact on clinical trials that use quantitative parametric ADC maps in diagnostic and treatment response metrics, and will supply a method for standardization of ADC map values across different clinical MRI platforms.

### Supplemental Materials

Supplemental Appendix:
