# Supplementary material for: QIN DAWG Validation of Gradient Nonlinearity Bias Correction Workflow for Quantitative Diffusion-Weighted Imaging in Multicenter Trials
Source: Tomography. 2016 Dec;2(4):396–405. doi: 10.18383/j.tom.2016.00214 (PMC5241082; doi:10.18383/j.tom.2016.00214)
Supplement: Supplemental Appendix: [file tom-00214-16-s001.pdf]

QIN Data Acquisition Work Group
DWI Gradient Non Linearity (GNL) Correction Validation Project
FBIRN PHANTOM DWI TEST INSTRUCTIONS
Version 20160303

Introduction:

GNL introduces a significant spatial bias in apparent diffusion coefficient (ADC) values. In the DAWG "phase 1" project, GNL bias was characterized for several MRI systems. In this "phase 2" project, GNL bias will be mitigated using corrector functions derived from "phase 1" data. A simple phantom having an unknown ADC will be scanned near isocenter where GNL bias is small thereby determining "true ADC" for the phantom. The phantom will then be displaced in the bore creating heavily-biased DWI. Only biased DWI data will be sent the University of Michigan for blinded correction. Correction performance metrics will be based on statistical comparison of biased and bias-corrected ADC histograms relative to true ADC.

Objectives:

1. For several representative MRI systems (characterized during "phase 1" GNL demo project), measure apparent diffusion coefficient (ADC) of a uniform (agar gel) phantom at isocenter and two semi-arbitrary off-center locations.
2. Apply pre-defined system-specific GNL correctors (previously obtained in "phase 1" using an ice water phantom) to biased ADC maps of another phantom to demonstrate that corrections are generally applicable to an independent objects and ADC value ranges.

Experiment Overview:

1. Use spherical gel phantom (e.g. FBIRN) to provide uniformity and avoid swirling media. Before scanning, let this phantom stay in the scan room for >24hrs to insure thermal equilibrium.
2. Use torso coils and largest field-of-view allowed by the system (e.g., 500x500mm) to encompass all three planned phantom positions. For ALL DWI scans, **have the DWI prescription remain centered in RL and SI** directions so the system does not offset the scan table from isocenter. Do not change FOV throughout this experiment and keep a single landmark when repositioning.
3. For the first (reference) scan ("**Position 1"**), place the phantom close to isocenter in RL and AP directions, and define the landmark centered on the phantom. Survey and plan "coronal" DWI scan to cover phantom, and tailor the shim volume to the phantom. For all DWI, provide each individual diffusion-encoding direction image, as well as the combined "trace" DWI.
4. Perform 3-orthogonal axes DWI with diffusion encoding in "lab X, Y, Z" directions using parameters specified in Table 1. **Use DSE-DWI sequence variant (if available).** If your system is able to acquire three orthogonal DWI in "non-lab axes, also acquire and save these DWI.
5. For the **"Position 2"**, pull the table out enough to gain access to the phantom. Move the phantom superiorly by 120 to 150mm within the coil. You may have to place an additional small water bottle near isocenter (original landmark) so the system is able to tune center frequency. Use original "reference" landmark, when moving table back to scan position. Acquire another survey scan so the shim volume is tailored to the new phantom location. Acquire another 3-orthogonal "lab-frame" DWI scan **without changing FOV and holding the center of the FOV at RL=0 and SI=0**. If your system is able to acquire three orthogonal DWI in non-lab frame, also acquire these DWI.
6. For the **"Position 3"**, pull the table as in step 2, but put the phantom on an elevation prop to offset the phantom anteriorly by 70 to 100mm, while still maintaining 120 to 150mm superior offset. Elevate the anterior torso coil accordingly. You can add arbitrary left or right offset (<100mm), as well. Repeat survey scan. **Only move the prescription of slices anteriorly to encompass the phantom, but keep the center of the FOV at RL=0 and SI=0.** Tailor the shim volume to the phantom at Position 3. Acquire another 3-orthogonal "lab-frame" DWI scan. If your system is able to acquire three orthogonal DWI in non-lab frame, also acquire these DWI.
7. Locally archive all images, but only upload DWI DICOM for off-center ("Position 2 and 3" only) to University of Michigan "M-Box". Do not upload the reference DWI series ("Position 1") for analysis, but generate ADC maps locally and perform ROI measurements close to isocenter to record unbiased ADC value for your system.

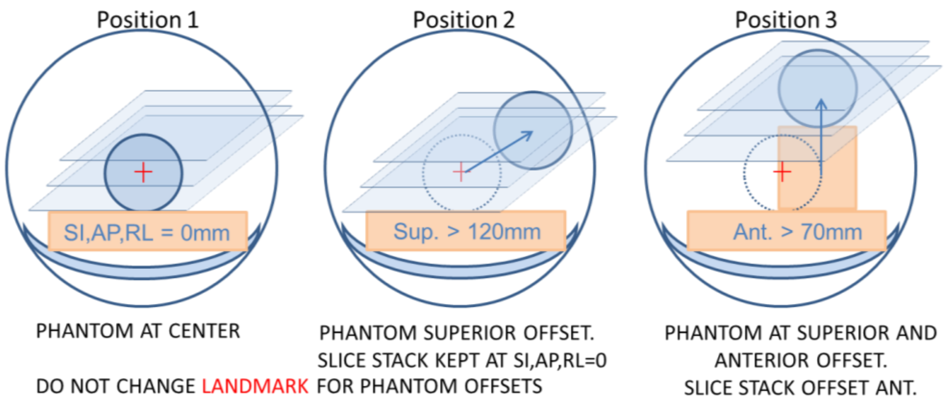

Figure 1: Schematic of FBIRN phantom positioning

Phantom Preparation Procedure:

1. ***Contents:*** FBIRN phantom contains 1.5% agar gel in a spherical container, diameter~170mm.
2. ***Set For Thermal Equilibrium:*** Store phantom in the scan room for >24 hours before scanning.
3. ***Elevation Props:*** Find suitable dense pads or other supports to hold the phantom elevated anteriorly by 70-100mm for "position 3". Set these props aside until phantom is moved to position 3.

Exam Demographics:

Start a "New Patient/Exam" using "Head First, Supine" convention, torso coil. Place the phantom on a thin foam pad directly on the scan table or torso coil. Center the phantom in RL direction and set landmark on the center of the phantom.

Series Acquired:

1. **Position 1 Survey:** Acquire orthogonal stacks in scout/survey using a large (~500x500mm) FOV; keep "image shutter" off to visualize the full FOV.
2. **Position 1 DWI:** See Table 1 for parameter settings. Place center of coronal slice stack at RL=0 and SI=0, but adjust stack AP offset to encompass the phantom. If possible, tailor shim volume to the phantom and use your best shim procedure. Acquire Position 1 DWI according to Table 1 settings using three-orthogonal diffusion encoding in lab frame. If possible, repeat for three-orthogonal axes in non-lab frame.
3. **Position 2 Survey:** Move phantom to position 2 and re-survey. Keep the survey FOV centered at SI=0 so the table does not offset relative to landmark.
4. **Position 2 DWI:** See Table 1 for parameter settings. Keep center of coronal slice stack at RL=0 and SI=0, but adjust stack AP offset to encompass the phantom. If possible, tailor shim volume to the phantom and use your best shim procedure. Use "full prescan preparation" and acquire Position 2 DWI according to Table 1 settings using three-orthogonal diffusion encoding in lab frame. If possible, repeat for three-orthogonal axes in non-lab frame.
5. **Position 3 Survey:** Move phantom to position 3 and re-survey. Keep the survey FOV centered at SI=0 so the table does not offset relative to landmark.
6. **Position 3 DWI:** See Table 1 for parameter settings. Keep center of coronal slice stack at RL=0 and SI=0, but adjust stack AP offset to encompass the phantom. If possible, tailor shim volume to the phantom and use your best shim procedure. Use "full prescan preparation" and acquire Position 3 DWI according to Table 1 settings using three-orthogonal diffusion encoding in lab frame. If possible, repeat for three-orthogonal axes in non-lab frame.

**Table 1. DWI Spherical (FBIRN) Phantom Scan Parameters**			
Field Strength	1.5T or 3T	DWI Sequence	Single-Shot EPI **Double Spin Echo** (DSE preferred, if available, or SSE, otherwise)
Receiver Coil	Torso Array	TR (ms)	≥4000
FOV (mm)	(450-500) x (450-500) keep FOV centered at RL=0 and SI=0	TE (ms)	min or 80-130
Acquisition Matrix	(100-128)x(100-128) [a]	Half-scan, Partial-Fourier, Frac-NEX	No [a]
Reconstruction Matrix	256 x 256 [b]	Number of Gradient Directions [c]	3 ortho-axes in LAB frame and (if possible) non-LAB frame
Orientation	Coronal, no obliquity (FoldOver, Phase=RL; Freq=SI)	Freq Enc Bandwidth per Acq Pixel (Hz) [d]	max
Quantity of Slices	29	Parallel Imaging	NO
Slice Thickness (mm)	5	bvalues (s/mm^2)[e]	0, 500, 1000
Gap (mm)	1	# Signal Averages	8
Image Filtering (eg.SCIC/CLEAR)	Off (e.g., use CLASSIC)	Fat Suppression	Optional
Shim	Use best shim method with volume tailored to phantom	Prescan	Use “full-preparation” for each DWI series

a) Some systems may require “half-scan”, “partial-Fourier”, or “fractional-NEX” for DWI. It is preferred to acquire the full phase-encode matrix by setting half-scan/ partial-Fourier/Fractional-NEX to “No” or at least as close to 1 as possible.
b) Interpolate image matrix to 256 x 256. Some systems may do this automatically.
c) Acquire DWI along three orthogonal LAB and (if possible) non-LAB axes so that direction-specific DWIs are generated. Isotropic or trace diffusion weighted images are optional.
d) Frequency encoding bandwidth may not be under full operator control. If possible use “maximum bandwidth”, or equivalently “minimum fat shift per pixel..
e) It is preferred to acquire all b-values in a single series.

Data Transfer:
Send an email to Tom at tlchenev@med.umich.edu and he will send you an invite to upload your DICOM images to his “M-Box” account. It is preferred to have all your images one zip file.

With questions, contact:

Thomas L. Chenevert: tlchenev@med.umich.edu ; 734-936-8866
Dariya Malyarenko: dariya@med.umich.edu ; 734-647-5532
